# Errors in Figures 2, 3, and 4 and Funding/Support

**DOI:** 10.1001/jamanetworkopen.2023.37926

**Published:** 2023-09-28

In the Consensus Statement titled “Management of Acetaminophen Poisoning in the US and Canada: A Consensus Statement,”^1^ published August 8, 2023, the 3 reference citations in the Figure 2 caption were incorrect; “acetaminophen” was incorrectly spelled in Figure 3; the flow chart in Figure 4 had a line of incorrect information regarding ingestion periods; and the funding source was described inaccurately and with an incorrect grant number in the Funding/Support section. This article has been corrected.^1^
